# System justification and democracy: Is liberal democracy part of the status quo?

**DOI:** 10.1111/bjso.70059

**Published:** 2026-02-25

**Authors:** Salvador Vargas Salfate, Rebecca Scheffauer, Homero Gil de Zúñiga

**Affiliations:** ^1^ Department of Psychology University of Illinois Urbana‐Champaign Champaign Illinois USA; ^2^ Democracy Research Unit, Political Science University of Salamanca Salamanca Spain; ^3^ Fil Production & Media Studies Pennsylvania State University University Park Pennsylvania USA; ^4^ Facultad de Comunicación y Letras Universidad Diego Portales Santiago Chile

**Keywords:** liberal democracy, political institutions, status quo, system justification

## Abstract

Research has conceptualized system justification as an overall perception of legitimacy of the status quo. However, there is mixed evidence to determine whether individuals construe political systems and values that uphold them as part of such status quo. We reasoned that if individuals construe the status quo as encompassing the political system and its values in the United States, system justification should predict support for current political institutions and liberal democracy. Relying on a representative survey and an experiment (*N* = 1994), we found that system justification was related to support for current institutions but not liberal democracy principles, even when making salient different components of the status quo (i.e. economic inequality and liberal democracy). Results suggest that researchers studying legitimacy of intergroup settings or political institutions should measure legitimacy of those institutions rather than general perceptions of fairness, as individuals might not construe the status quo as encompassing those institutions.

## INTRODUCTION

Research has explored how individuals legitimize a variety of intergroup settings and institutions, such as gender hierarchies (e.g. Yeung et al., 2014), economic inequality (e.g. Brown‐Iannuzzi et al., 2021) and political systems (e.g. Badaan et al., 2020). In that context, a strand of the literature has attempted to analyse broader perceptions of fairness and legitimacy that would include these different intergroup settings and institutions (e.g. Jost et al., 2015; Sidanius et al., 2004). Consistent with this attempt, researchers have used broad measures to assess support for the status quo. One prominent example is the system justification scale (Kay & Jost, 2003). This scale measures perceived fairness and legitimacy of the national *economic* system (e.g. *Everyone has a fair shot at wealth and happiness*), national *political* system (e.g. *In general, the American political system operates as it should*), attachment to the nation (e.g. *The United States is the best country in the world to live in*) and of the overall society (or even national meritocracy; e.g. *Society is set up so that people usually get what they deserve*).^1^ In that sense, all items are portrayed in a general way with the explicit purpose of measuring overall support for the status quo (Owuamalam et al., 2019)—or the defence and rationalization of current social, political and economic arrangements or systems (Jost, 2019). This example raises a broader theoretical and empirical question we attempt to partially address in this research: What intergroup settings and institutions are part of what people consider to be the status quo? One component of the status quo is its political institutions and principles. As we review below, there is heterogeneous evidence in the literature on whether *people* construe politics as part of the status quo. We focus on the specific case of liberal democracy because of its relevance to most Western countries—and particularly the United States (U.S.), which is the context where we tested our hypotheses.

## INCONSISTENT EVIDENCE ON THE ASSOCIATION BETWEEN SYSTEM JUSTIFICATION AND POLITICS

Politics is an inherent part of the status quo (Jost, 2019). Therefore, support for the status quo should encompass support for current political systems, such as liberal democracy in the United States. However, previous studies have provided heterogeneous evidence when examining whether people construe the status quo in this way when using broad measures of support for the status quo such as the system justification scale.

A first group of studies provides support for the idea that individuals construe political systems as part of the status quo, showing that individuals higher in system justification reject politicians and parties that challenge the political status quo. For example, Langer et al. (2023) found that individuals higher in system justification preferred to vote more for mainstream (vs. antiestablishment) parties in France, Germany and the United Kingdom (U.K.). In the same sense, Vasilopoulos and Jost (2020) found that system justification was associated with less support for populism—a concept usually defined by a strong Manichaeism and antiestablishment ideology that divides society between dualistic groups such as ‘us’, the good people (e.g. citizens) versus ‘them’, the corrupt elites (e.g. politicians) (Jay et al., 2019; Marcos‐Marne et al., 2023; Oxendine, 2019). Populism has received increasing attention in recent years with Donald Trump getting elected as president, or the Brexit as examples of ‘backlash against established political elites and institutions’ (Speed & Mannion, 2017, p. 249).

A second group of studies shows evidence that does not support the interpretation that individuals construe the political systems as part of the status quo. Several studies have shown that system justification was associated with more support for candidates opposing incumbents in countries such as the United States and Argentina (Azevedo et al., 2017; Jost et al., 2017; Monteith & Hildebrand, 2019). For example, people high in system justification supported Trump more in the 2016 election (Azevedo et al., 2017; Monteith & Hildebrand, 2019)—when the political status quo was represented by the Obama administration (i.e. when the incumbent was Obama). Trump was an outsider to the political institutions, as he was an entrepreneur without experience as a politician that endorsed a somewhat antiestablishment agenda. Many of Trump's voters came from a place of dissatisfaction with previous governments and saw promise in a candidate not so deeply ingrained in the traditional set of party politics and networks (Speed & Mannion, 2017). Candidates like Trump tend to rally against liberal elites as well as established independent media institutions and judiciaries (Isaac, 2017), thus further subverting the status quo—as it has also been observed in other cultural contexts (Dijkstra et al., 2020; Kyle & Gultchin, 2018). If system justification would be associated with more support for current political systems, then people scoring high in this scale would have rejected (and not supported) Trump in 2016.

In summary, previous studies have provided heterogeneous evidence for the idea that support for the status quo encompasses support for current political systems. Therefore, in this research, we provide a direct empirical approach to address the questions raised for these different groups of studies. More specifically, we argue that if the current political systems are construed as part of the status quo, a measure of support for the status quo (e.g. system justification) should be associated with more support for liberal democracy in the United States Accordingly, not finding such an association would yield strong doubts that people construe political systems as part of the status quo.

We focused on the United States because it is a useful case study. The United States is generally described as a liberal democracy by international indexes assessing the state of political systems across the globe (Freedom House, 2025; Herre et al., 2025). In other words, the status quo in the United States can be described as encompassing liberal democracy (Freedom House, 2023, 2025). Furthermore, this description is similar to those observed for other contexts (e.g. Europe, Oceania, some South American countries), which includes the free right to vote, freedom of association and the implementation of legislative check and balances. Moreover, political changes in the United States are also similar to changes in other countries. The (re)election of Trump as president represents a contrast with the previous status quo, as he advocates against liberal elites and established independent media institutions and judiciaries (Isaac, 2017). In this way, studying the United States could also be informative for other context with similar political characteristics.

## ATTITUDES TOWARDS LIBERAL DEMOCRACY

Liberal democracy encompasses different components. One common component of liberal democracy definitions is the ability to hold those in power accountable for their actions (Huntington, 1993; Schumpeter, 1950). Accountability can take different forms depending on whether actions originate from citizenship or from other governing bodies and political institutions, and it might vary across countries. While political accountability and its outcomes may differ across political contexts, it remains an important and integral part of democracy (de la Torre, 2014).

Another distinguishing component of liberal democracy refers to liberties and freedoms granted to individuals, including entitlement to participation, either directly or via representatives, and people's freedom to their opinions (Bollen, 1986, 1993; Dahl, 2008). This component also includes freedom to practice religious beliefs or freedom of education without indoctrination, as well as political rights such as fair elections, safeguards against corruption, and transparency and openness of government actions (Freedom House, 2023). One relevant consideration is that these freedoms typically have boundaries in liberal democracies. Certain laws and regulations restrict personal freedom to ensure other people's freedoms and rights. These restrictions include, for example, legislation against hate speech (Kulenović, 2023) or censorship of the internet to prevent harmful content (Busch et al., 2018; Caesmann et al., 2024).

In applied research, these components of liberal democracy have been analysed through objective indicators such as voter turnout or how legislative bodies are composed (Cutright, 1969; Lerner, 1958; Vanhanen, 1990). Another approach has focused on subjective indicators through experts' evaluations, which include freedom of the media, fairness of elections or political liberties (Bollen & Paxton, 2000; Burkhart & Lewis‐Beck, 1994; Muller & Seligson, 1994). In this research, we focus on people's attitudes towards liberal democracy instead of on the objective state of this type of political system. We define attitudes towards liberal democracy as those attitudes consistent with the aforementioned conceptualization of liberal democracy in this study. Specifically, we conceive attitudes consistent with liberal democracy as those attitudes supporting institutional accountability, respect for liberties and freedom, and participation in institutional and non‐institutional politics.

## THE PRESENT RESEARCH

In this research, we examine whether empirical evidence is consistent with the idea that people construe politics as part of the status quo. Given that previous studies have provided contradictory ideas suggesting that people might not always construe politics as part of the status quo (e.g. Azevedo et al., 2017; Jost et al., 2017; Langer et al., 2023; Monteith & Hildebrand, 2019; Vasilopoulos & Jost, 2020), we empirically revisit the assumption that an overall measure of support for the status quo (i.e. system justification scale; Kay & Jost, 2003) should be associated with more support for liberal democracy in the United States. Even though finding these correlations does not directly imply that people construe politics as part of the status quo, the lack of consistent associations between these constructs casts doubts on this assumption—as we precisely show across two studies.

In this research, we distinguish between *current* status quo (e.g. political institutions) and the *principles* underlying the status quo (e.g. respect for civil liberties). If people construe politics as part of the status quo, system justification should be associated with perceiving that political institutions work satisfactorily and with support for principles that uphold these institutions. In Study 1, we relied on a representative survey from the United States and tested whether system justification is associated with less political dissatisfaction and more political trust (Buer & Fatke, 2014), as those can be conceived as indicators of how people perceive the current political status quo (e.g. Brandt, 2013; Owuamalam et al., 2023). Indeed, political trust is conceived as an evaluation of political institutions based on their performance, according to what people consider political institutions ought to do (Hetherington, 1998). Relatedly, political dissatisfaction reflects a negative perception of how political institutions work addressing people's needs, which could even elicit behaviours inconsistent with supporting political institutions (e.g. protest behaviour) (Christensen, 2016). For these reasons, if the status quo encompasses politics, we expected that supporting the status quo (i.e. higher values in system justification) would correspond with more political trust and less political dissatisfaction. Importantly, dissatisfaction with current institutions is different from rejecting the principles underlying the political systems (Ferrin & Kriesi, 2016; Kaltwasser & Hauwaert, 2020; Mudde & Kaltwasser, 2012). For this reason, we also tested whether support for the status quo would be associated with support for political repression, as rejecting repression represents a relevant component of liberal democracy *principles* (Gibson, 1988).

In Study 1, we also conducted the main analyses examining our ideas after adjusting for authoritarianism, political ideology and political partisanship. Authoritarianism encompasses respect for traditions, submission to authorities and rejection of dissenters (Altemeyer, 1998; Duckitt & Bizumic, 2013), whereas political ideology (i.e. conservatism) encompasses support for inequality and rejection of change (Jost et al., 2003). In the United States, political partisanship (i.e. republican, democrat) also conveys meaningful information about people's views around the status quo, as party preferences have become strongly aligned with ideology over time (Abramowitz & Saunders, 2008). In this way, adjusting for these variables allows examining the association between support for the status quo and support for liberal democracy, isolating this association from the influence of ideological variables that are typically associated with general support for the status quo (Vargas‐Salfate et al., 2018).

In Study 2, we conducted a preregistered experiment to further address our research question, making more explicit the distinction between support for *current* political institutions and liberal democracy *principles*. We also made salient different components of the status quo in the United States to examine whether making salient that politics is part of the status quo would lead to stronger associations between support for the status quo and support for liberal democracy.

Across both studies, we only present the results directly testing our ideas in the main text. Full results can be found in the Online Supplementary Material (OSM). All datasets, analysis code and research material are available at https://osf.io/knrxe. Study 2 was preregistered and can be accessed at https://osf.io/34v8c.

## STUDY 1

In Study 1, we used secondary data to measure support for current democratic principles and practices as well as support for the status quo (i.e. system justification). We included political trust, political dissatisfaction and support for political repression, which are commonly used in the political science literature (e.g. Marcos‐Marne et al., 2023). We used all these indicators to examine whether people supporting more the status quo (i.e. high in system justification) exhibit favourable attitudes towards democracy, which is the status quo in the United States. In subsequent analyses we controlled for authoritarianism, political ideology and political partisanship to discard that any association between support for the status quo and attitudes towards democracy would be explained by right‐wing traits and ideologies, rather than perceptions of legitimacy of the status quo (see Caricati, 2019; Langer et al., 2023; Vesper et al., 2022).

### Method

#### Participants

A total of 1365 participants were recruited by IPSOS in June 2019 to respond to a questionnaire about politics and media through Qualtrics (for more information, see Gil de Zúñiga & Goyanes, 2023). Demographics of our sample are comparable to US census data (see Table S1 in the OSM).

#### Measures

All variables were coded such that higher values express higher presence of the construct used to label the different scales.

##### System justification

We used three items from the System justification scale (Kay & Jost, 2003) (i.e. *In general, I find society to be fair*, *In general, our political system operates as it should* and *Everyone in my country has a fair shot at wealth and happiness*), with responses ranging from 1 (*strongly disagree*) to 10 (*strongly agree*). This scale was reliable in our sample (*α* = .79).

##### Political dissatisfaction

We included four items to indirectly measure political dissatisfaction. These items expressed the level of concern with respect to *the extent of crime*, *the extent of unemployment*, *the difference between rich and poor* and *the cost of living* (Marcos‐Marne et al., 2023). All responses ranged from 1 (*not at all concerned*) to 10 (*very concerned*). These items formed a highly reliable scale (*α* = .78).

##### Political trust

We included two items in which participants rated their trust towards the *Government* and the *Political system in the United States*. Responses ranged from 1 (*not at all*) to 10 (*completely*). These items were significantly associated, *r* (1321) = .78, *p* < .001 and formed a highly reliable scale (*α* = .87, *Spearman‐Brown Reliability* = .87).

##### Support for political repression

We included three items to measure support for political repression (i.e. *Some people should be banned or prohibited in politics*, *Some groups should be banned from public employment* and *Some people should be prohibited from running as candidates or holding public office*). Responses to these items ranged from 1 (*strongly disagree*) to 10 (*strongly agree*), which formed a highly reliable scale (*α* = .85).

##### Authoritarianism

We included three items from the Right‐wing authoritarianism scale (Altemeyer, 1996) (i.e. *Obedience and respect for authority are the most important virtues children should learn*, *Our country needs a powerful leader, in order to destroy the radical and immoral elements in society today*, and *In these troubled times, laws have to be enforced without mercy, especially when dealing with the agitators and revolutionaries who are ‘stirring things up’*). Responses to these items ranged from 1 (*strongly disagree*) to 10 (*strongly agree*), which formed a highly reliable scale (*α* = .81).

##### Political partisanship

We included a single item asking participants whether they identified as *Republican*, *Democrat* or *Independent* in a scale ranging from 0 (*Strong Democrat*) to 10 (*Strong Republican*), with the middle point of the scale 5 as *Independent* (Wojcieszak & Price, 2010).

##### Political ideology

Finally, we included two items to measure ideology on *political issues* and *economic issues* (Pratto et al., 1997). Responses to these items ranged from 0 (*strong liberal*) to 10 (*strong conservative*). These items were significantly associated, *r* (1260) = .86, *p* < .001 and formed a highly reliable scale (*α* = .92, *Spearman‐Brown Reliability* = .92).

### Results and discussion

Descriptive statistics and correlations matrix are shown in Table 1. As expected by our theoretical argument, we found that higher scores in system justification corresponded with more political trust and less political dissatisfaction. However, inconsistent with our predictions, we found that system justification was not associated with support for political repression. We found similar results when controlling for authoritarianism, political partisanship and political ideology—with the exception that higher scores in system justification corresponded with less support for political repression, *b* = −.10, *SE* = .04, *p* = .010, 95% CI [−.15, −.02], b* = −.08 (all details of these models can be found in the OSM, Tables S2–S4).

**TABLE 1:** Correlations matrix (Study 1).

	*M*	*SD*	1	2	3	4	5	6	7
1. System justification	4.89	2.18	–						
2. Political trust	4.18	2.35	.51***	–					
3. Political dissatisfaction	6.83	2.04	−.08**	.05	–				
4. Political repression	5.72	2.72	.04	.02	.27***	–			
5. Authoritarianism	5.82	2.38	.38***	.17***	.19***	.31***	–		
6. Political partisanship	5.04	3.00	.24***	.02	−.13***	.02	.29***	–	
7. Political ideology	5.46	2.80	.31***	.04	−.13***	.03	.39***	.72***	–

** *p* < .01.

*** *p* < .001.

In summary, Study 1 showed partial support for the idea that support for the general status quo predicts support for the political status quo in the United States. We found that higher scores in system justification were associated with more political trust and less political dissatisfaction, which indicates that people might be construing how political institutions work as part of the status quo. Consistently, we also found that higher scores in system justification were associated with less support for political repression. However, this pattern of findings only emerged when controlling for additional theoretically relevant covariates. From a methodological perspective, this finding could be a suppressor effect—a positive association that becomes negative only after adjusting for covariates (Cheung & Lau, 2007). From a conceptual perspective, this finding could suggest that only when comparing people with similar political ideology and authoritarianism, supporting the status quo is associated with less support for political repression—as higher scores in authoritarianism were significantly associated with both more support for political repression and support for the status quo. This interpretation would also indicate that measures of supporting the status quo could encompass different constructs that contaminate their associations with relevant outcomes such as political repression.

Importantly political repression might be construed as promoting conformity to the *current* political system, but it is inconsistent with democratic *principles*. Therefore, we conducted a preregistered study more explicitly distinguishing between support for *current* democratic institutions and liberal democracy *principles*. We also sought to address some limitations in Study 1, such as the use of indirect indicators to assess how people evaluate political institutions' work (i.e. political dissatisfaction).

## STUDY 2

In Study 2, we sought to extend results from Study 1. We used a methodological paradigm that resembles studies that have found that making salient people's relationships increases the association between relationship satisfaction and life satisfaction (Strack et al., 1988)—as people's responses to life satisfaction become anchored on their relationships. The general idea is that support for the status quo (i.e. system justification) should predict more support for components of the status quo, such as liberal democracy and economic inequality. More specifically, these associations should be stronger when making salient these components of the status quo, in comparison to a control condition where these components are not made salient. Importantly, we also distinguished between *current* status quo (e.g. political institutions) and the *principles* underlying the status quo (e.g. respect for civil liberties). As we did in Study 1, we focused on the case of liberal democracy and compared it with economic inequality as well as a control condition where we did not make salient any aspect of the status quo (i.e. showing participants how to prepare a salad). We added economic inequality for two reasons. First, it provides a control condition for the liberal democracy condition where we made salient a different aspect of the status quo. And second, we sought to extend our theoretical argument to a domain highly studied in the context of support for the status quo (Goudarzi et al., 2022) using measures as system justification (e.g. Goudarzi et al., 2020; Wakslak et al., 2007). We included measures of perception and legitimation of economic inequality, as current social norms in the economic domain in Western countries consider economic inequality to be acceptable when they arise as a consequence of differences in skills, qualifications and effort (Batruch et al., 2022; Trump, 2020). When examining whether making salient that economic inequality is part of the status quo leads to stronger associations between support for the status quo and support for economic inequality, the liberal democracy condition serves also as a control condition.

Specifically, we preregistered the following hypotheses:

Hypothesis 1 Making salient liberal democracy as part of the status quo in the U.S. (vs. economic inequality and control) will lead to *stronger* positive associations between general system justification and support for liberal democracy.

Hypothesis 2 Making salient economic inequality as part of the status quo in the U.S. (vs. economic inequality and control) will lead to *stronger* positive associations between general system justification and support for economic inequality.

In addition, as system justification measures support for the status quo, when not making salient any aspect of the status quo, this scale would still be associated with support for liberal democracy and economic inequality, which is summarized in the following hypotheses:

Hypothesis 3a When not making salient any aspect of the status quo (i.e., in a control condition), system justification will be *positively* associated with support for liberal democracy.

Hypothesis 3b When not making salient any aspect of the status quo (i.e., in a control condition), system justification will be *positively* associated with support for economic inequality.

We included both measures of support for the current status quo and the principles underlying it. Most of the literature using broad measures of support for the status quo (e.g. system justification scale) has focused on support for current inequalities and political institutions. Empirical research has related system justification to specific perceptions of extant inequality as fair and support for current political institutions, which have been operationalized as support for affirmative action (Phelan & Rudman, 2011), rejection of feminism (Yeung et al., 2014), among other variables. However, there is also research within this approach that has highlighted that system justification might be associated with the principles expressed through the status quo, such as the case of liberal values in France (Langer et al., 2020). Therefore, in this research we also sought to address which specific facets of the status quo are related to system justification (i.e. its *current* form or its *principles*).

Finally, it is important to acknowledge that there is a strong conceptual overlap between system justification and all measures related to support for liberal democracy and economic inequality—as they are facets of the status quo. In that sense, our hypotheses are focused on how making salient aspects of the status quo can lead to differences in the associations between system justification and these facets of the status quo.

### Method

#### Participants

We conducted an a priori statistical power analysis to determine the sample size. Specifically, we conducted 1000 Monte Carlo simulations in *R* (R Core Team, 2022) for a linear regression model consistent with our methodological design (i.e. one continuous predictor, two dummy predictors indicating the three conditions in our experiment and the interactions between the continuous predictor and conditions). We specified all regression coefficients to be equal to .15, as this is the effect size for the association between system justification and conservatism observed in previous studies (Azevedo et al., 2017). We used this association, as we could not identify previous associations between system justification and support for current democracy or liberal democracy principles. This analysis revealed that a sample size of 600 participants would provide a statistical power between .82 and .84 for the interaction terms in the linear regression model. We then recruited 660 US residents through CloudResearch (Litman et al., 2017) in May 2023. As we had preregistered, we excluded 31 participants because they failed at least one of the attention checks we included, which asked participants to indicate the topic of the text they had to read in the condition they were assigned (i.e. *political institutions in the United States*, *economic inequality in the United States* or *how to prepare a salad*) and to respond *yes* to a blank box. This led us to have a sample size of 629 participants. Most participants were men (*N* = 317) or women (*N* = 306), and only six indicated other in the gender identification variable (*M*_age_ = 44.31, *SD* = 13.00). The majority of participants self‐identified as *White/Caucasian/European* (*N* = 514), followed by *Black/African‐American* (*N* = 63), *Asian* (*N* = 38), *Hispanic/Latino/South American* (*N* = 27), *Aboriginal/Native* (*N* = 5) and *Middle Easterner* (*N* = 2).

#### Procedure

Participants were invited to participate in a study about social issues in the United States. After consenting to participate, they were randomly assigned to one of the conditions in our experiment. In the *liberal democracy condition*, participants read that the status quo in the United States comprises the political system:

The status quo in the United States is defined by different elements. One of such elements is the political system. In the United States, political institutions ought to follow certain principles. These principles include accountability (i.e., institutions must be responsible for their acts), and respect for liberties and freedom such as people have the right to participate in politics, among others.

In the *economic inequality condition*, participants read that the status quo in the United States comprises the economic system:

The status quo in the United States is defined by different elements. One of such elements is the economic system. In the United States, the economy is organized by certain principles. These principles include respect for private property, the right to earn more income when people get more education, etc. This results in economic inequality that ought to be a consequence of individual merit such as effort, education, skills, etc.

Participants in the *neutral control condition* read a generic description of a salad recipe of similar length than the text in the other conditions. Then, all participants were asked to select the topic they read about in the first part of the experiment as part of one of the attention checks. Participants next completed all the scales in the study, in a random order and finally completed some demographic questions.

#### Measures

All measures were assessed on a scale ranging from 1 (*strongly disagree*) to 7 (*strongly agree*).

##### System justification

We included the full version of the system justification scale (Kay & Jost, 2003). This scale was highly reliable in our sample (*α* = .89).

##### Support for current democracy

We included the Democratic System Justification Scale (Rutto et al., 2014) adapted to the United States to measure support for current democracy. This scale comprises eight items (e.g. *In general, the American political system operates as it should*), and it was highly reliable in our sample (*α* = .85).

##### Support for liberal democracy principles

We conducted a pilot study among 213 U.S. undergraduates and asked them to rate how consistent were several items with the definition of the liberal democracy principles we used in our research (i.e. institutional accountability, respect for liberties and freedom, and participation in politics). We selected eight items that corresponded to those that were rated by these participants as most consistent with these principles (e.g. *The media should report the news without censorship*, *The Congress should discuss the laws proposed by the President*, *People should be able to run for public office if they want*). This Pilot Study is reported in the OSM. The resulting scale was highly reliable in the sample that completed Study 2 (*α* = .82).

##### Legitimation of inequality

We included four items from the Subjective Inequality Scale (factor unfairness beliefs; Schmalor & Heine, 2022) (e.g. *It is not fair if there are large differences in income between the rich and the poor*). We recoded these values such that higher values would express higher legitimation of economic inequality. This scale was highly reliable in our sample (*α* = .89).

##### Perceived inequality

We included four items from the Subjective Inequality Scale (factor subjective inequality; Schmalor & Heine, 2022) (e.g. *Almost all the money that is earned goes to only a few people*). This scale was highly reliable in our sample (*α* = .92).

### Results and discussion

We conducted a series of exploratory and preliminary analyses comparing all variables by condition. Results from these analyses are fully reported in the OSM (Tables S5 and S6).

As preregistered, we regressed legitimation of inequality, perceived inequality, support for *current* democracy and support for liberal democracy *principles* on system justification (grand‐mean centred), liberal democracy condition (liberal democracy condition coded as 0.5, all other conditions coded as −0.5/2), economic inequality condition (economic inequality condition coded as 0.5, all other conditions coded as −0.5/2) and the two‐way interactions between system justification and conditions.^2^

Results are fully reported in the OSM (Tables S7–S10) and indicate that participants higher in system justification legitimated inequality more, *b* = .59, b* = .47, *p* < .001, 95% CI [.50, .67], supported more *current* democracy, *b* = .77, b* = .85, *p* < .001, 95% CI [.73, .81] and perceived less inequality *b* = −.62, b* = −.50, *p* < .001, 95% CI [−.70, −.54]. System justification, however, was unrelated to support for liberal democracy *principles*, *b* = −.04, b* = −.06, *p* = .126, 95% CI [−.09, .01]. Importantly, none of the interactions between system justification and conditions (factor effects coded) were significantly associated with the dependent variables, all *p*s ≥ .115. In other words, making salient specific aspects of the status quo did not lead to higher associations between system justification and support for those aspects of the status quo—which is not consistent with Hypotheses 1 and 2. Consistently with Hypotheses 3a and 3b, simple slope analyses indicate that when not making salient any aspect of the status quo (i.e. in the control condition), participants higher in system justification supported more *current* democracy, *b* = .75, b* = .83, *p* < .001, 95% CI [.68, .81], legitimated more inequality, *b* = .58, b* = .47, *p* < .001, 95% CI [.43, .73] and perceived less inequality, *b* = −.61, b* = −.50, *p* < .001, 95% CI [−.76, −.47]. Inconsistently with Hypothesis 3, however, when not making salient any aspect of the status quo (i.e. in the control condition), system justification was not associated with support for liberal democracy *principles*, *b* = −.03, b* = −.04, *p* = .542, 95% CI [−.12, .06].^3^

In summary, in Study 2, we found that regardless of which aspect of the status quo was made salient, people supporting the status quo more (i.e. high in system justification) legitimated more economic inequality, perceived less economic inequality and supported more *current* democracy. However, support for the status quo was unrelated to support for liberal democracy *principles*, casting doubts on the idea that *all* aspects of politics are construed as part of the status quo.

## GENERAL DISCUSSION

Previous research has explored how and why individuals justify the status quo, including social systems to which they belong or legitimize intergroup relations and institutions. However, less research has explored what constitutes the status quo for individuals. We sought to explore whether people construed the current political systems, and specifically liberal democracy, as part of such a status quo. We tested these ideas using a commonly used measure of support for the status quo, such as the system justification scale (Jost, 2019; Kay & Jost, 2003). We decided to focus on this specific component of the status quo for three main reasons. First, liberal democracy in the United States, the cultural setting where we explored our ideas, is a core component of the political system (Freedom House, 2023, 2025). Second, there is mixed evidence regarding whether individuals construe their political systems as part of the status quo. System justification predicted less support for antiestablishment parties and populist politicians in countries like France, Germany and the United Kingdom. (Langer et al., 2023; Vasilopoulos & Jost, 2020). However, system justification predicted Trump support when Obama was president (Azevedo et al., 2017; Monteith & Hildebrand, 2019) and support for right‐wing candidates in Argentina when the president was leftist (Jost et al., 2017). And third, recent research has suggested that normative values that constitute political systems can be construed as part of the status quo—as in the case of France (Langer et al., 2020).

Across two studies, we found partial support for the idea that individuals might construe the status quo as encompassing the current political system. We found that higher values in system justification corresponded with more political trust and less political dissatisfaction, but also more support for repression—which is a dimension not necessarily consistent with liberal democracy *principles* (Dahl, 2008). We observed these associations after adjusting for authoritarianism, political ideology (i.e. conservatism) and political partisanship. In other words, these associations are unique to support for the status quo and cannot be attributed to other belief systems that are typically associated with this construct (e.g. Vargas‐Salfate et al., 2018). Furthermore, regardless of which aspect of the status quo we made salient in an experimental design, system justification predicted higher support for *current* democracy, but it was unrelated to support for liberal democracy *principles*.

These results suggest that at least in the United States, individuals might construe the status quo as encompassing the specific institutions and policies implemented by the political system *in their current form*. However, the normative values that uphold these systems are shared by people scoring high and low in system justification. Indeed, the means of the measure of support for liberal democracy *principles* showed a ceiling effect, as it was very close to the highest end of the scale and the standard deviation was the lowest among those included in Study 2.^4^ In other words, what differentiates people high and low in system justification is their evaluations of how political institutions work, and not necessarily the support for main principles they are expected to represent. Complementary, these results also suggest that liberal democracy principles are central to most people's views of politics. In this way, our research also suggests that normative contexts should be considered when studying why and how people legitimate the status quo (for a related discussion see Rubin et al., 2023).

Our results parallel findings in the economic domain, where the endorsement of meritocratic values (i.e. prescriptive meritocracy) are highly consensual, but individuals tend to differ in the belief that their societies fulfil those principles (i.e. descriptive meritocracy) (Davey et al., 1999; Son Hing et al., 2011). Our results suggest that normative values associated with liberal democracy are highly consensual in the United States and that the differences between individuals high (vs. low) in system justification is in how they evaluate the way in which institutions function and how they work consistently with those values. This liberal consensus could be a consequence of social norms prescribing liberal democracy values and free market (over other principles) to a greater extent than in other countries. This type of argument has received more empirical attention in post‐communist countries, but it could also be extended to the United States (e.g. Evans & Kelley, 2017). Importantly, these processes might also vary across countries, as in France indirect evidence suggests that people high in system justification support the normative values that uphold the French democracy (Langer et al., 2020)—which in turn highlights the relevance of considering contextual factors in political psychology.

Importantly, this liberal consensus does not necessarily imply that people have similar construals of liberal democracy principles. Previous studies have indicated that system justification is associated with more authoritarian attitudes (e.g. Vargas‐Salfate et al., 2018). In that sense, our study suggests that people with different scores in system justification might agree that liberal democracy in the United States is important, but they differ in what democracy means to them. In fact, it is also possible that people could have different construals of repression. Even in liberal democracies, freedom has boundaries to protect other people's freedom and safety (e.g. Busch et al., 2018; Kulenović, 2023). In that sense, future research should develop more fine grained measures of political repression to better understand the association between general support for the status quo and support for political repression.

Relatedly, and considering the empirical evidence around system justification and support for political institutions, one of the main implications of our research is that overall measures of support for the status quo might be construed in different ways by individuals. This is similar to the case of other social‐psychological constructs that are theorized to encompass global preferences for intergroup hierarchies, such as social dominance orientation (Schmitt et al., 2003). For that reason, researchers addressing the legitimacy of specific institutions, intergroup relations, or hierarchies should prefer to use measures assessing the legitimacy of the specific institutions, intergroup settings, etc. they are studying, instead of relying on general measures of support for the status quo.

### Limitations and future research directions

Our research has limitations that should be addressed in future research. We used a brief priming procedure to make salient certain aspects of the status quo in our experimental design in Study 2—which led us to, for example, focus on specific components of liberal democracy while excluding others (e.g. minority rights, free and fair elections). Participants had to read a short paragraph that explained how the status quo in the United States encompassed economic inequality or liberal democracy. As our manipulation was brief and implemented over an online experiment, an alternative explanation for the results that did not support part of our hypotheses is that the manipulation did not prime participants with the specific contents of the status quo we made salient. We cannot rule out this alternative explanation, as we did not pretest our manipulation in a different sample. Furthermore, we included a control condition that was unrelated to the main topic of study, which could increase demand characteristics leading to noise in the data (Boot et al., 2013). In that sense, future research should address this limitation by testing whether our hypotheses would be supported when using different procedures.

A second limitation is related to the measures used across both studies. In Study 1, we mostly relied in short versions of the scales. For instance, system justification and authoritarianism were assessed with only three items per scale, and ideology only included items assessing conservatism in political and economic issues. This decision is consistent with previous studies relying on large representative datasets not directly designed for the purpose of the current study (e.g. Kesberg et al., 2024; Vargas‐Salfate et al., 2018). To partially address this limitation, we conducted all analyses from Study 2 using the same items measuring system justification as in Study 1. Both versions of the scale were highly correlated, *r* (627) = .94, *p* < .001 and results did not meaningfully change (all analyses are reported in the OSM, Tables S16–S19). We only found that this measure of system justification predicted support for liberal democracy principles, b* = −.09, *p* = .019, but the magnitude of this association was smaller than the rest of the significant associations reported in this article and emerged only after computing multiple comparisons—and indeed this association would not be considered as significant when using Bonferroni correction for multiple comparisons (i.e. *α* = .05/4 = .013). For that reason, this association should be cautiously interpreted. In addition, in Study 2, we did not include measures of ideology or authoritarianism, as our design was mostly focused on exploring associations between system justification and political outcomes when making salient different aspects of the status quo. Future research should test these ideas after adjusting for theoretically relevant covariates such as ideology. Furthermore, across both studies, we assessed support for the status quo using the system justification scale (Kay & Jost, 2003). We decided to focus on this specific measure as it is widely used in the context of studies addressing legitimacy of the status quo (Jost, 2019). In that sense, future research should address the external validity of our results by testing whether they generalize to other measures of support for the status quo.

Finally, in Study 2, we included two control conditions: economic inequality and a neutral control condition. Economic inequality is a more specific condition than liberal democracy. As such, our results could be influenced by this difference. Future studies should test our ideas using economic system (i.e. capitalism) as a control condition, in addition to neutral control conditions.

## CONCLUDING REMARKS

Different studies assessing the legitimacy and fairness of the status quo have used the concept and measure of system justification. However, previous results were inconsistent when attempting to determine whether individuals construe the status quo as encompassing the political system and its core normative values—which in the case of the United States would correspond to liberal democracy. Across two studies, we found evidence supporting the idea that current political institutions, but not the core normative values, are construed as part of the status quo. Indeed, liberal democracy *principles* appeared to be highly endorsed and consensual in our study.

## AUTHOR CONTRIBUTIONS

**Salvador Vargas Salfate:** Conceptualization; investigation; writing – original draft; methodology; writing – review and editing; formal analysis. **Rebecca Scheffauer:** Conceptualization; investigation; writing – original draft; writing – review and editing. **Homero Gil de Zúñiga:** Conceptualization; investigation; writing – original draft; methodology; writing – review and editing; supervision.

## CONFLICT OF INTEREST STATEMENT

The authors have no conflicts of interest to declare.

## Supporting information


**Table S1.** Demographic Profile of Study Survey (Study 1), Other Comparable US Survey and the US Census.
**Table S2**. Results From Main Analyses (Study 1): Political Trust.
**Table S3**. Results From Main Analyses (Study 1): Political Dissatisfaction.
**Table S4**. Results From Main Analyses (Study 1): Political repression.
**Table S5**. Comparisons Across Condition (Study 2).
**Table S6**. Descriptive Statistics and Correlations Matrix (Study 2).
**Table S7**. Results From Main Analyses (Study 2): Legitimation of inequality.
**Table S8**. Results From Main Analyses (Study 2): Support for current inequality.
**Table S9**. Results From Main Analyses (Study 2): Support for current democracy.
**Table S10**. Results From Main Analyses (Study 2): Support for liberal democracy principles.
**Table S11**. Results With 7‐item Measure of System Justification (Study 2): Legitimation of inequality.
**Table S12**. Results With 7‐item Measure of System Justification (Study 2): Support for current inequality.
**Table S13**. Results With 7‐item Measure of System Justification (Study 2): Support for current democracy.
**Table S14**. Results With 7‐item Measure of System Justification (Study 2): Support for liberal democracy principles.
**Table S15**. Results for Liberal Democracy Principles Transformed.
**Table S16**. Results With 3‐item Measure of System Justification (Study 2): Legitimation of inequality.
**Table S17**. Results With 3‐item Measure of System Justification (Study 2): Support for current inequality.
**Table S18**. Results With 3‐item Measure of System Justification (Study 2): Support for current democracy.
**Table S19**. Results With 3‐item Measure of System Justification (Study 2): Support for liberal democracy principles.
**Table S20**. Results from Pilot Study.

## Data Availability

Data to replicate the findings will be available upon reasonable request to the PI of the project (HGZ). Codes with analyses can be found at https://osf.io/knrxe.

## Footnotes

^1^
We use status quo and system as interchangeable (e.g. Jost, 2019).

^2^
As we included several dependent variables, the false discovery rate is likely to be higher than 5% (*α* = .05). When considering a Bonferroni correction for α to control the false discovery rate (i.e. *α* = .05/4), all significant effects in this section are still below this threshold.

^3^
As our measure of support for current democracy contained an item very similar to one of the items in the system justification scale (i.e. *In general, the American political system operates as it should*), we repeated all analyses excluding this item from the system justification scale. None of the interpretations provided in the main text changed when excluding this item. These results are fully reported in the OSM (Tables S11–S14).

^4^
We repeated the main analyses using the natural logarithm of support for liberal democracy *principles* as the dependent variable. We did not find differences with our previous analyses. These results are fully reported in the OSM (Table S15).
