# Supplementary material for: System justification and democracy: Is liberal democracy part of the status quo?
Source: Br J Soc Psychol. 2026 Feb 25;65(2):e70059. doi: 10.1111/bjso.70059 (PMC12936248; doi:10.1111/bjso.70059)
Supplement: Supplementary file 1 — Table S1. Demographic Profile of Study Survey (Study 1), Other Comparable US Survey and the US Census. Table S2. Results From Main Analyses (Study 1): Political Trust. Table S3. Results From Main Analyses (Study 1): Political Dissatisfaction. Table S4. Results From Main Analyses (Study 1): Political repression. Table S5. Comparisons Across Condition (Study 2). Table S6. Descriptive Statistics and Correlations Matrix (Study 2). Table S7. Results From Main Analyses (Study 2): Legitimation of inequality. Table S8. Results From Main Analyses (Study 2): Support for current inequality. Table S9. Results From Main Analyses (Study 2): Support for current democracy. Table S10. Results From Main Analyses (Study 2): Support for liberal democracy principles. Table S11. Results With 7‐item Measure of System Justification (Study 2): Legitimation of inequality. Table S12. Results With 7‐item Measure of System Justification (Study 2): Support for current inequality. Table S13. Results With 7‐item Measure of System Justification (Study 2): Support for current democracy. Table S14. Results With 7‐item Measure of System Justification (Study 2): Support for liberal democracy principles. Table S15. Results for Liberal Democracy Principles Transformed. Table S16. Results With 3‐item Measure of System Justification (Study 2): Legitimation of inequality. Table S17. Results With 3‐item Measure of System Justification (Study 2): Support for current inequality. Table S18. Results With 3‐item Measure of System Justification (Study 2): Support for current democracy. Table S19. Results With 3‐item Measure of System Justification (Study 2): Support for liberal democracy principles. Table S20. Results from Pilot Study. [file BJSO-65-0-s001.docx]

**System Justification and Democracy: Is Liberal Democracy Part of the *Status Quo*? Online Supplementary Material**

**Contents**

[Description of Sample (Study 1) 4](#_Toc207378755)

[Table S1. Demographic Profile of Study Survey (Study 1), Other Comparable US Survey, and the US Census 4](#_Toc207378756)

[Results From Main Analyses Controlling for Covariates (Study 1) 6](#_Toc207378757)

[Table S2. Results From Main Analyses (Study 1): Political Trust 7](#_Toc207378758)

[Table S3. Results From Main Analyses (Study 1): Political Dissatisfaction 8](#_Toc207378759)

[Table S4. Results From Main Analyses (Study 1): Political repression 9](#_Toc207378760)

[Exploratory Analyses: Comparisons Across Conditions (Study 2) 10](#_Toc207378761)

[Table S5. Comparisons Across Condition (Study 2) 10](#_Toc207378762)

[Exploratory Analyses: Descriptive Statistics and Correlations Among Variables (Study 2) 11](#_Toc207378763)

[Table S6. Descriptive Statistics and Correlations Matrix (Study 2) 11](#_Toc207378764)

[Results From Main Analyses (Study 2) 12](#_Toc207378765)

[Table S7. Results From Main Analyses (Study 2): Legitimation of inequality 13](#_Toc207378766)

[Table S8. Results From Main Analyses (Study 2): Support for current inequality 14](#_Toc207378767)

[Table S9. Results From Main Analyses (Study 2): Support for current democracy 15](#_Toc207378768)

[Table S10. Results From Main Analyses (Study 2): Support for liberal democracy principles 16](#_Toc207378769)

[Results When Using 7-item Measure of System Justification (Study 2) 17](#_Toc207378770)

[Table S11. Results With 7-item Measure of System Justification (Study 2): Legitimation of inequality 18](#_Toc207378771)

[Table S12. Results With 7-item Measure of System Justification (Study 2): Support for current inequality 19](#_Toc207378772)

[Table S13. Results With 7-item Measure of System Justification (Study 2): Support for current democracy 20](#_Toc207378773)

[Table S14. Results With 7-item Measure of System Justification (Study 2): Support for liberal democracy principles 21](#_Toc207378774)

[Results With Support for Liberal Democracy Principles Transformed (Study 2) 22](#_Toc207378775)

[Table S15. Results for Liberal Democracy Principles Transformed 23](#_Toc207378776)

[Results When Using 3-item Measure of System Justification (Study 2) 24](#_Toc207378777)

[Table S16. Results With 3-item Measure of System Justification (Study 2): Legitimation of inequality 25](#_Toc207378778)

[Table S17. Results With 3-item Measure of System Justification (Study 2): Support for current inequality 26](#_Toc207378779)

[Table S18. Results With 3-item Measure of System Justification (Study 2): Support for current democracy 27](#_Toc207378780)

[Table S19. Results With 3-item Measure of System Justification (Study 2): Support for liberal democracy principles 28](#_Toc207378781)

[Pilot Study to Develop Measure of Support for Liberal Democracy Principles 29](#_Toc207378782)

[Material 29](#_Toc207378783)

[Table S20. Results from Pilot Study 33](#_Toc207378784)

# Description of Sample (Study 1)

A full description of the sample used in Study 1 and how it compares to other relevant studies is shown in Table S1.

## Table S1. Demographic Profile of Study Survey (Study 1), Other Comparable US Survey, and the US Census

|  | Study Survey Jun. 2019 | | General Social Survey (GSS), 2018 | U.S. Census Bureau (Estimates, 2019) |
| --- | --- | --- | --- | --- |
|  | | (%) | (%) | (%) |
| *Gender:* | |  |  |  |
| Female | | 53.2 | 55.2 | 51.5 |
| Male | | 46.8 | 44.8 | 48.5 |
| *Race / Ethnicity:* | |  |  |  |
| White | | 75.2 | 71.2 | 77.5 |
| Black or African American | | 10.1 | 16.4 | 13.0 |
| Hispanic or Latino | | 6.5 | 5.4 | 14.6 |
| Asian | | 5.6 | 3.1 | 6.1 |
| *Age:* | |  |  |  |
| 18-35 | | 32.3 | 5.5 | 30.6 |
| 36-55 | | 39.7 | 33.2 | 36.7 |
| Older than 55 | | 28.0 | 38.2 | 32.7 |
| *Education:* | |  |  |  |
| High school or less | | 35.2 | 61.3 | 38.9 |
| Some college | | 25.0 | 8.3 | 27.8 |
| Bachelor’s degree or more | | 39.8 | 30.3 | 33.3 |
| *Household Income:* | |  |  |  |
| Less than $15,000 | | 11.6 | 21.2 | 9.1 |
| $15,000 to $24,999 | | 9.8 | 12.7 | 8.0 |
| $25,000 to $49,999 | | 21.1 | 30.8 | 20 |
| $50,000 to $99,999 | | 33.3 | 24.0 | 28.8 |
| $100,000 to $149,999 | | 15.9 | 3.3 | 15.5 |
| $150,000 to $199,999 | | 4.8 | 7.9 | 8.3 |
| $200,000 or more | | 3.5 | 0.0 | 10.3 |

*Note:* Percentages are calculated for population 18 years old and over. In the second column, ‘Race’ (block #2) was measured by respondents’ first mention, ‘Some college’ (block #4) includes those who reported ‘Junior College’ as their highest degree in the GSS, and Household Income (block #5) was measured in inflation-adjusted constant dollars (See GSS Methodological Reports). The third column (blocks #1-4) shows official estimates of the resident population by single year for the United States (2019). For education attainment, Census figures also refer to population age 18 and older, and “some college” also includes those with an associate’s degree. Household-level income data (block #5) in the third column is based on information from the Current Population Survey Annual Social and Economic Supplements (CPS ASEC). Source (third column): U.S. Census Bureau, Population Division.

# Results From Main Analyses Controlling for Covariates (Study 1)

Results from main analyses controlling for covariates in Study 1 are presented in Tables S2-S4.

## Table S2. Results From Main Analyses (Study 1): Political Trust

|  | *b* | *b** | *se* | *t* | *p* | *95% CI (b*)* | | *b* | *b** | *se* | *t* | *p* | *95% CI* | | *VIF* |
| --- | --- | --- | --- | --- | --- | --- | --- | --- | --- | --- | --- | --- | --- | --- | --- |
|  |  |  |  |  |  | *LB* | *UB* |  |  |  |  |  | *LB* | *UB* |  |
| Intercept | 1.46 |  | 0.14 | 10.30 | <.001 |  |  | 1.82 |  | 0.18 | 10.32 | <.001 |  |  |  |
| System justification | 0.56 | 0.52 | 0.03 | 21.30 | <.001 | 0.47 | 0.56 | 0.60 | 0.55 | 0.03 | 20.86 | <.001 | 0.50 | 0.60 | 1.21 |
| Authoritarianism | |  |  |  |  |  |  | 0.02 | 0.02 | 0.03 | 0.63 | 0.592 | -0.04 | 0.07 | 1.31 |
| Political partisanship | | |  |  |  |  |  | -0.04 | -0.05 | 0.03 | -1.55 | 0.122 | -0.12 | 0.01 | 2.09 |
| Political ideology | |  |  |  |  |  |  | -0.08 | -0.10 | 0.03 | -2.64 | 0.008 | -0.17 | -0.02 | 2.30 |
| *F* test | *F* (1, 1256) = 452, *p* < .001, $R^{2}$ = .27 | | | | | | | *F* (4, 1253) = 123, *p* < .001, $R^{2}$ = .28 | | | | | | | |
| *F* change |  | | | | | | | *F* (3) = 9.7, *p* < .001 | | | | | | | |

Note: *b* indicates unstandardized coefficient and *b** indicates standardized coefficient.

## Table S3. Results From Main Analyses (Study 1): Political Dissatisfaction

|  | *b* | *b** | *se* | *t* | *p* | *95% CI (b*)* | | *b* | *b** | *se* | *t* | *p* | *95% CI* | | *VIF* |
| --- | --- | --- | --- | --- | --- | --- | --- | --- | --- | --- | --- | --- | --- | --- | --- |
|  |  |  |  |  |  | *LB* | *UB* |  |  |  |  |  | *LB* | *UB* |  |
| Intercept | 7.27 |  | 0.14 | 51.83 | <.001 |  |  | 6.77 |  | 0.17 | 40.26 | <.001 |  |  |  |
| System justification | -0.09 | -0.09 | 0.03 | -3.34 | <.001 | -0.13 | -0.03 | -0.15 | -0.16 | 0.03 | -5.45 | <.001 | -0.19 | -0.09 | 1.21 |
| Authoritarianism | |  |  |  |  |  |  | 0.30 | 0.35 | 0.03 | 11.48 | <.001 | 0.25 | 0.35 | 1.30 |
| Political partisanship | | |  |  |  |  |  | -0.06 | -0.08 | 3.00 | -0.19 | 0.029 | -0.14 | -0.01 | 2.10 |
| Political ideology | |  |  |  |  |  |  | -0.12 | -0.16 | 0.03 | -3.98 | <.001 | -0.21 | -0.07 | 2.30 |
| *F* test | *F* (1, 1258) = 11.2, *p* < .001, $R^{2}$ = .01 | | | | | | | *F* (4, 1255) = 41.5, *p* < .001, $R^{2}$ = .12 | | | | | | | |
| *F* change |  | | | | | | | *F* (3) = 52.7, *p* < .001 | | | | | | | |

Note: *b* indicates unstandardized coefficient and *b** indicates standardized coefficient.

## Table S4. Results From Main Analyses (Study 1): Political repression

|  | *b* | *b** | *se* | *t* | *p* | *95% CI (b*)* | | *b* | *b** | *se* | *t* | *p* | *95% CI (b*)* | | *VIF* |
| --- | --- | --- | --- | --- | --- | --- | --- | --- | --- | --- | --- | --- | --- | --- | --- |
|  |  |  |  |  |  | *LB* | *UB* |  |  |  |  |  | *LB* | *UB* |  |
| Intercept | 5.46 |  | 0.19 | 28.89 | <.001 |  |  | 4.17 |  | 0.23 | 18.41 | <.001 |  |  |  |
| System justification | 0.05 | 0.04 | 0.04 | 1.48 | 0.140 | -0.02 | 0.11 | -0.10 | -0.08 | 0.04 | -2.59 | 0.010 | -0.15 | -0.02 | 1.21 |
| Authoritarianism | |  |  |  |  |  |  | 0.44 | 0.38 | 0.03 | 12.55 | <0.001 | 0.37 | 0.51 | 1.30 |
| Political partisanship | | |  |  |  |  |  | <0.01 | <0.01 | 0.04 | 0.01 | 0.994 | -0.09 | 0.09 | 2.10 |
| Political ideology | |  |  |  |  |  |  | -0.09 | -0.10 | 0.04 | -2.39 | 0.017 | -0.20 | -0.02 | 2.30 |
| *F* test | *F* (1, 1259) = 2.2, *p* = .138, $R^{2}$ < .01 | | | | | | | *F* (4, 1256) = 40.1, *p* < .001, $R^{2}$ = .11 | | | | | | | |
| *F* change |  | | | | | | | *F* (3) = 52.7, *p* < .001 | | | | | | | |

Note: *b* indicates unstandardized coefficient and *b** indicates standardized coefficient.

# Exploratory Analyses: Comparisons Across Conditions (Study 2)

We compared all our measures across conditions, which is shown in Table S5. We found significant differences when predicting legitimation of inequality. Pairwise post-hoc analyses using Tukey HSD suggested that participants in the economic inequality condition legitimized economic inequality more than those in the liberal democracy condition (*p* = .016). We also found significant differences when predicting perception of inequality, but pairwise post-hoc analyses did not reach conventional levels of significance when comparing each condition to each other (*p* ≥ .056). None of the other variables were affected by condition.

## Table S5. Comparisons Across Condition (Study 2)

|  | Liberal democracy condition | | Economic inequality condition | | Control condition | | Mean comparisons |
| --- | --- | --- | --- | --- | --- | --- | --- |
|  | *M* | *SD* | *M* | *SD* | *M* | *SD* |  |
| System justification | 3.72 | 1.32 | 3.78 | 1.33 | 3.70 | 1.28 | *F* (2, 626) = 0.19, *p* = .829*,* $\eta^{2}$ < .01 |
| Legitimation of inequality | 2.98 | 1.60 | 3.43 | 1.69 | 3.06 | 1.62 | *F* (2, 626) = 4.48, *p* = .012*,* $\eta^{2}$ *=* .01 |
| Perception of inequality | 4.57 | 1.55 | 4.26 | 1.66 | 4.62 | 1.62 | *F* (2, 624) = 3.10, *p* = .046*,* $\eta^{2}$ *=* .01 |
| Support for *current* democracy | 3.62 | 1.20 | 3.63 | 1.16 | 3.65 | 1.18 | *F* (2, 626) = 0.02, *p* = .977*,* $\eta^{2}$ < .01 |
| Support for liberal democracy *principles* | 5.97 | 0.87 | 5.93 | 0.87 | 5.98 | 0.77 | *F* (2, 625) = 0.23, *p* = .797*,* $\eta^{2}$ < .01 |

# Exploratory Analyses: Descriptive Statistics and Correlations Among Variables (Study 2)

We also explored the associations between our constructs, which are shown in Table S6. Overall, system justification was associated with greater legitimation of economic inequality and support for *current* democracy, but with lower perception of economic inequality. Importantly, system justification was not associated with support for *liberal* democracy principles.

## Table S6. Descriptive Statistics and Correlations Matrix (Study 2)

|  | M | SD | 1 | 2 | 3 | 4 | 5 |
| --- | --- | --- | --- | --- | --- | --- | --- |
| 1. System justification | 3.73 | 1.31 | 1 |  |  |  |  |
| 2. Legitimation of inequality | 3.16 | 1.65 | .47*** | 1 |  |  |  |
| 3. Perception of inequality | 4.48 | 1.62 | -.51*** | -.65*** | 1 |  |  |
| 4. Support for *current* democracy | 3.63 | 1.18 | .85*** | .29*** | -.36*** | 1 |  |
| 5. Support for liberal democracy *principles* | 5.96 | 0.84 | -.06 | <.01 | -.06 | -.12** | 1 |

* *p* < .05

** *p* <. 01

*** *p* < .001

# Results From Main Analyses (Study 2)

Results from main analyses in Study 2 are shown in Tables S7-S10.

## Table S7. Results From Main Analyses (Study 2): Legitimation of inequality

|  | *b* | *b** | *se* | *t* | *p* | *95% CI* | |
| --- | --- | --- | --- | --- | --- | --- | --- |
|  |  |  |  |  |  | *LB* | *UB* |
| Intercept | 3.15 |  | 0.06 | 55.10 | <.001 | 3.04 | 3.27 |
| Liberal democracy condition | -0.12 | -0.03 | 0.19 | -0.63 | 0.528 | -0.49 | 0.25 |
| System justification | 0.59 | 0.47 | 0.04 | 13.39 | <.001 | 0.50 | 0.67 |
| Economic inequality condition | 0.43 | 0.09 | 0.19 | 2.30 | 0.022 | 0.06 | 0.79 |
| Liberal democracy condition × System justification | -0.22 | -0.06 | 0.14 | -1.53 | 0.126 | -0.50 | 0.06 |
| Economic inequality condition × System justification | 0.22 | 0.06 | 0.14 | 1.58 | 0.115 | -0.05 | 0.50 |
| *F* test | *F* (5, 623) = 40.50, *p* < .001, $R^{2}$ = .25 | | | | | | |

Note: *b* indicates unstandardized coefficient and *b** indicates standardized coefficient.

## Table S8. Results From Main Analyses (Study 2): Support for current inequality

|  | *b* | *b** | *se* | *t* | *p* | *95% CI* | |
| --- | --- | --- | --- | --- | --- | --- | --- |
|  |  |  |  |  |  | *LB* | *UB* |
| Intercept | 4.49 |  | 0.06 | 81.17 | <.001 | 4.38 | 4.59 |
| Liberal democracy condition | -0.05 | -0.01 | 0.18 | -0.27 | 0.783 | -0.41 | 0.31 |
| System justification | -0.62 | -0.50 | 0.04 | -14.71 | <.001 | -0.70 | -0.54 |
| Economic inequality condition | -0.4 | -0.09 | 0.18 | -2.25 | 0.025 | -0.76 | -0.05 |
| Liberal democracy condition × System justification | 0.18 | 0.05 | 0.14 | 1.27 | 0.206 | -0.10 | 0.45 |
| Economic inequality condition × System justification | -0.21 | -0.06 | 0.14 | -1.53 | 0.126 | -0.48 | 0.06 |
| *F* test | *F* (5, 621) = 47.00, *p* < .001, $R^{2}$= .27 | | | | | | |

Note: *b* indicates unstandardized coefficient and *b** indicates standardized coefficient.

## Table S9. Results From Main Analyses (Study 2): Support for current democracy

|  | *b* | *b** | *se* | *t* | *p* | *95% CI* | |
| --- | --- | --- | --- | --- | --- | --- | --- |
|  |  |  |  |  |  | *LB* | *UB* |
| Intercept | 3.63 |  | 0.02 | 147.81 | <.001 | 3.59 | 3.68 |
| Liberal democracy condition | -0.05 | -0.02 | 0.08 | -0.65 | 0.514 | -0.21 | 0.11 |
| System justification | 0.77 | 0.85 | 0.02 | 40.92 | <.001 | 0.73 | 0.81 |
| Economic inequality condition | -0.10 | -0.03 | 0.08 | -1.23 | 0.218 | -0.26 | 0.06 |
| Liberal democracy condition × System justification | 0.08 | 0.03 | 0.06 | 1.22 | 0.223 | -0.05 | 0.20 |
| Economic inequality condition × System justification | 0.01 | <0.01 | 0.06 | 0.10 | 0.923 | -0.11 | 0.13 |
| *F* test | *F* (5, 623) = 335.00, *p* < .001, $R^{2}$= .73 | | | | | | |

Note: *b* indicates unstandardized coefficient and *b** indicates standardized coefficient.

## Table S10. Results From Main Analyses (Study 2): Support for liberal democracy principles

|  | *b* | *b** | *se* | *t* | *p* | *95% CI* | |
| --- | --- | --- | --- | --- | --- | --- | --- |
|  |  |  |  |  |  | *LB* | *UB* |
| Intercept | 5.96 |  | 0.03 | 177.98 | <.001 | 5.89 | 6.03 |
| Liberal democracy condition | <0.01 | <0.01 | 0.11 | -0.03 | 0.979 | -0.21 | 0.21 |
| System justification | -0.04 | -0.06 | 0.03 | -1.53 | 0.126 | -0.09 | 0.01 |
| Economic inequality condition | -0.06 | -0.03 | 0.11 | -0.57 | 0.571 | -0.27 | 0.15 |
| Liberal democracy condition × System justification | -0.03 | -0.02 | 0.08 | -0.40 | 0.693 | -0.20 | 0.13 |
| Economic inequality condition × System justification | -0.01 | -0.01 | 0.08 | -0.17 | 0.866 | -0.18 | 0.15 |
| *F* test | *F* (5, 622) = 0.59, *p* = .707, $R^{2}$ < .01 | | | | | | |

Note: *b* indicates unstandardized coefficient and *b** indicates standardized coefficient.

# Results When Using 7-item Measure of System Justification (Study 2)

We excluded the item that directly measured support for the political system from the system justification scale and repeated all main analyses. These results are shown in Tables S11-S14.

## Table S11. Results With 7-item Measure of System Justification (Study 2): Legitimation of inequality

|  | *b* | *b** | *se* | *t* | *p* | *95% CI* | |
| --- | --- | --- | --- | --- | --- | --- | --- |
|  |  |  |  |  |  | *LB* | *UB* |
| Intercept | 3.16 |  | 0.06 | 55.79 | <.001 | 3.04 | 3.27 |
| Liberal democracy condition | -0.14 | -0.03 | 0.19 | -0.74 | 0.458 | -0.50 | 0.23 |
| System justification | 0.62 | 0.49 | 0.04 | 14.15 | <.001 | 0.53 | 0.70 |
| Economic inequality condition | 0.41 | 0.09 | 0.18 | 2.25 | 0.025 | 0.05 | 0.77 |
| Liberal democracy condition × System justification | -0.20 | -0.06 | 0.14 | -1.41 | 0.16 | -0.48 | 0.08 |
| Economic inequality condition × System justification | 0.24 | 0.07 | 0.14 | 1.67 | 0.095 | -0.04 | 0.52 |
| *F* test | *F* (5, 623) = 44.7, *p* < .001, $R^{2}$ = .26 | | | | | | |

Note: *b* indicates unstandardized coefficient and *b** indicates standardized coefficient.

## Table S12. Results With 7-item Measure of System Justification (Study 2): Support for current inequality

|  | *b* | *β* | *se* | *t* | *p* | *95% CI* | |
| --- | --- | --- | --- | --- | --- | --- | --- |
|  |  |  |  |  |  | *LB* | *UB* |
| Intercept | 4.49 |  | 0.05 | 82.24 | <.001 | 4.38 | 4.59 |
| Liberal democracy condition | -0.03 | -0.01 | 0.18 | -0.17 | 0.869 | -0.38 | 0.32 |
| System justification | -0.65 | -0.52 | 0.04 | -15.46 | <.001 | -0.73 | -0.57 |
| Economic inequality condition | -0.39 | -0.09 | 0.18 | -2.20 | 0.028 | -0.74 | -0.04 |
| Liberal democracy condition × System justification | 0.17 | 0.05 | 0.14 | 1.20 | 0.232 | -0.11 | 0.44 |
| Economic inequality condition × System justification | -0.23 | -0.07 | 0.14 | -1.66 | 0.097 | -0.50 | 0.04 |
| *F* test | *F* (5, 621) = 51.6, *p* < .001, $R^{2}$ = .29 | | | | | | |

Note: *b* indicates unstandardized coefficient and *b** indicates standardized coefficient.

## Table S13. Results With 7-item Measure of System Justification (Study 2): Support for current democracy

|  | *b* | *b** | *se* | *t* | *p* | *95% CI* | |
| --- | --- | --- | --- | --- | --- | --- | --- |
|  |  |  |  |  |  | *LB* | *UB* |
| Intercept | 3.63 |  | 0.03 | 137.91 | <.001 | 3.58 | 3.68 |
| Liberal democracy condition | -0.08 | -0.02 | 0.09 | -0.91 | 0.363 | -0.25 | 0.09 |
| System justification | 0.75 | 0.83 | 0.02 | 36.83 | 0.156 | 0.71 | 0.79 |
| Economic inequality condition | -0.11 | -0.03 | 0.09 | -1.29 | 0.198 | -0.28 | 0.06 |
| Liberal democracy condition × System justification | 0.08 | 0.03 | 0.07 | 1.15 | 0.252 | -0.05 | 0.21 |
| Economic inequality condition × System justification | <0.01 | <0.01 | 0.07 | 0.02 | 0.986 | -0.13 | 0.13 |
| *F* test | *F* (5, 623) = 272, *p* < .001, $R^{2}$ = .69 | | | | | | |

Note: *b* indicates unstandardized coefficient and *b** indicates standardized coefficient.

## Table S14. Results With 7-item Measure of System Justification (Study 2): Support for liberal democracy principles

|  | *b* | *b** | *se* | *t* | *p* | *95% CI* | |
| --- | --- | --- | --- | --- | --- | --- | --- |
|  |  |  |  |  |  | *LB* | *UB* |
| Intercept | 5.96 |  | 0.03 | 177.78 | <.001 | 5.89 | 6.03 |
| Liberal democracy condition | <0.01 | <0.01 | 0.11 | -0.02 | 0.983 | -0.22 | 0.21 |
| System justification | -0.03 | -0.04 | 0.03 | -1.05 | 0.296 | -0.08 | 0.02 |
| Economic inequality condition | -0.06 | -0.03 | 0.11 | -0.57 | 0.567 | -0.28 | 0.15 |
| Liberal democracy condition × System justification | -0.03 | -0.02 | 0.08 | -0.34 | 0.733 | -0.2 | 0.14 |
| Economic inequality condition × System justification | -0.02 | -0.01 | 0.08 | -0.21 | 0.834 | -0.18 | 0.15 |
| *F* test | *F* (5, 622) = 0.34, *p* = .892, $R^{2}$ < .01 | | | | | | |

Note: *b* indicates unstandardized coefficient and *b** indicates standardized coefficient.

# Results With Support for Liberal Democracy Principles Transformed (Study 2)

We repeated the main analyses for support for liberal democracy *principles* in Study 2 because we found a ceiling effect. More specifically, we used this outcome variable after transforming it by computing its natural logarithm. Results from this model are shown in Table S15.

## Table S15. Results for Liberal Democracy Principles Transformed

|  | *b* | *b** | *se* | *t* | *p* | *95% CI* | |
| --- | --- | --- | --- | --- | --- | --- | --- |
|  |  |  |  |  |  | *LB* | *UB* |
| Intercept | 1.77 |  | 0.01 | 291.57 | <.001 | 1.76 | 1.79 |
| Liberal democracy condition | <0.01 | -0.01 | 0.02 | -0.21 | 0.834 | -0.04 | 0.03 |
| System justification | -0.01 | -0.07 | <0.01 | -1.66 | 0.097 | -0.02 | <0.01 |
| Economic inequality condition | -0.01 | -0.03 | 0.02 | -0.73 | 0.466 | -0.05 | 0.02 |
| Liberal democracy condition × System justification | -0.01 | -0.02 | 0.02 | -0.52 | 0.603 | -0.04 | 0.02 |
| Economic inequality condition × System justification | <0.01 | -0.01 | 0.02 | -0.18 | 0.855 | -0.03 | 0.03 |
| *F* test | *F* (5, 622) = 0.73, *p* = .599, $R^{2}$ = .01 | | | | | | |

Note: *b* indicates unstandardized coefficient and *b** indicates standardized coefficient.

# Results When Using 3-item Measure of System Justification (Study 2)

We used the same three items as in Study 1 to measure system justification. These results are shown in Tables S16-S19.

## Table S16. Results With 3-item Measure of System Justification (Study 2): Legitimation of inequality

|  | *b* | *b** | *se* | *t* | *p* | *95% CI* | |
| --- | --- | --- | --- | --- | --- | --- | --- |
|  |  |  |  |  |  | *LB* | *UB* |
| Intercept | 3.15 |  | 0.06 | 54.60 | <.001 | 3.04 | 3.27 |
| Liberal democracy condition | -0.10 | -0.02 | 0.19 | -0.53 | 0.596 | -0.47 | 0.27 |
| System justification | 0.48 | 0.45 | 0.04 | 12.74 | <.001 | 0.41 | 0.56 |
| Economic inequality condition | 0.45 | 0.10 | 0.19 | 2.41 | 0.016 | 0.08 | 0.82 |
| Liberal democracy condition × System justification | -0.21 | -0.07 | 0.13 | -1.65 | 0.100 | -0.45 | 0.04 |
| Economic inequality condition × System justification | 0.17 | 0.06 | 0.12 | 1.38 | 0.167 | -0.07 | 0.41 |
| *F* test | *F* (5, 623) = 37.5, *p* < .001, $R^{2}$ = .23 | | | | | | |

Note: *b* indicates unstandardized coefficient and *b** indicates standardized coefficient.

## Table S17. Results With 3-item Measure of System Justification (Study 2): Support for current inequality

|  | *b* | *b** | *se* | *t* | *p* | *95% CI* | |
| --- | --- | --- | --- | --- | --- | --- | --- |
|  |  |  |  |  |  | *LB* | *UB* |
| Intercept | 4.48 |  | 0.06 | 78.38 | <.001 | 4.37 | 4.60 |
| Liberal democracy condition | -0.06 | -0.01 | 0.19 | -0.34 | 0.73 | -0.43 | 0.30 |
| System justification | -0.47 | -0.45 | 0.04 | -12.6 | <.001 | -0.55 | -0.40 |
| Economic inequality condition | -0.43 | -0.09 | 0.19 | -2.31 | 0.021 | -0.79 | -0.06 |
| Liberal democracy condition × System justification | 0.21 | 0.07 | 0.12 | 1.65 | 0.099 | -0.04 | 0.45 |
| Economic inequality condition × System justification | -0.12 | -0.04 | 0.12 | -0.98 | 0.327 | -0.36 | 0.12 |
| *F* test | *F* (5, 621) = 35.5, *p* < .001, $R^{2}$ = .22 | | | | | | |

Note: *b* indicates unstandardized coefficient and *b** indicates standardized coefficient.

## Table S18. Results With 3-item Measure of System Justification (Study 2): Support for current democracy

|  | *b* | *b** | *se* | *t* | *p* | *95% CI* | |
| --- | --- | --- | --- | --- | --- | --- | --- |
|  |  |  |  |  |  | *LB* | *UB* |
| Intercept | 3.63 |  | 0.03 | 131.43 | <.001 | 3.58 | 3.69 |
| Liberal democracy condition | -0.03 | -0.01 | 0.09 | -0.28 | 0.779 | -0.20 | 0.15 |
| System justification | 0.62 | 0.81 | 0.02 | 34.51 | <.001 | 0.59 | 0.66 |
| Economic inequality condition | -0.07 | -0.02 | 0.09 | -0.78 | 0.437 | -0.25 | 0.11 |
| Liberal democracy condition × System justification | 0.08 | 0.04 | 0.06 | 1.33 | 0.183 | -0.04 | 0.20 |
| Economic inequality condition × System justification | -0.02 | -0.01 | 0.06 | -0.34 | 0.731 | -0.14 | 0.09 |
| *F* test | *F* (5, 623) = 239.00, *p* < .001, $R^{2}$ = .66 | | | | | | |

Note: *b* indicates unstandardized coefficient and *b** indicates standardized coefficient.

## Table S19. Results With 3-item Measure of System Justification (Study 2): Support for liberal democracy principles

|  | *b* | *b** | *se* | *t* | *p* | *95% CI* | |
| --- | --- | --- | --- | --- | --- | --- | --- |
|  |  |  |  |  |  | *LB* | *UB* |
| Intercept | 5.96 |  | 0.03 | 178.43 | <.001 | 5.89 | 6.03 |
| Liberal democracy condition | <0.01 | <0.01 | 0.11 | -0.03 | 0.974 | -0.22 | 0.21 |
| System justification | -0.05 | -0.09 | 0.02 | -2.34 | 0.019 | -0.09 | -0.01 |
| Economic inequality condition | -0.06 | -0.03 | 0.11 | -0.56 | 0.573 | -0.27 | 0.15 |
| Liberal democracy condition × System justification | 0.01 | 0.01 | 0.07 | 0.11 | 0.912 | -0.13 | 0.15 |
| Economic inequality condition × System justification | 0.02 | 0.01 | 0.07 | 0.29 | 0.768 | -0.12 | 0.16 |
| *F* test | *F* (5, 622) = 1.20, *p* = .707, $R^{2}$ = .01 | | | | | | |

Note: *b* indicates unstandardized coefficient and *b** indicates standardized coefficient.

# Pilot Study to Develop Measure of Support for Liberal Democracy Principles

We conducted a pilot study to select items to be included as indicators of support for current democracy principles. We asked 213 to indicate to which degree different items were consistent with the definition of several components of liberal democracy principles.

## Material

In this study, we are interested in developing a scale measuring support for liberal democracy principles. Based on a literature review, we identified three key dimensions of liberal democracy: institutional accountability, respect for liberties and freedom, and participation in politics. We will present a definition for each dimension and a series of items that we think might be indicators of these dimensions. We will ask you to rate how each of these items is consistent with those definitions. An item is consistent with a definition if it is appropriate to measure the proposed dimension. In other words, if an individual supports a given dimension (i.e., institutional accountability, respect for liberties and freedom, and participation in politics), then that individual would agree with the items selected to measure that dimension in a higher degree than an individual not agreeing with such dimension.

There is one exception for this criterion. There are items marked with an * that indicates that the item is reverse. This means that we expect people supporting any of the dimensions to score *lower* in these items in comparison with individuals not supporting these dimensions.

**Example**

We will illustrate the task with the following example based on meritocracy as a principle:

Meritocracy is defined as a justice principle according to which resources are allocated according to people’s effort, intelligence, and education.

Now, please rate how the following items are consistent with this definition. In other words, if an individual supporting meritocracy would score higher than an individual not supporting meritocracy. Use a scale from 1 (*not consistent at all with meritocracy*) to 7 (*highly consistent with meritocracy*).

1. The effort a worker puts into a job ought to be reflected in the size of a raise he or she receives.
2. * The effort a worker puts into a job ought **not** to be reflected in the size of a raise he or she receives.
3. The United States is the best country in the world to live in.

**Explanation of the example**

Remember the definition we provided: Meritocracy is defined as a justice principle according to which resources are allocated according to people’s effort, intelligence, and education.

In the previous example, these items were consistent with meritocracy because they were directly related to the definition we provided:

1. The effort a worker puts into a job ought to be reflected in the size of a raise he or she receives.
2. * The effort a worker puts into a job ought **not** to be reflected in the size of a raise he or she receives.

However, the following item was **not** consistent with meritocracy because it was not directly related to the definition we provided:

1. The United States is the best country in the world to live in.

[Attentional check]

Before completing the task, we want to be sure that you understood the instructions. Please, respond the following question:

What is the task we will ask you to complete?

1. State whether you agree or not with the principles of liberal democracy.
2. State whether you think the items presented are consistent with the principles of liberal democracy.

[Main task]

We identified three principles of liberal democracy: institutional accountability, respect for liberties and freedom, and participation in politics.

**Institutional accountability:** This dimension refers to the existence of mechanisms to hold authorities accountable for their decisions and to be able to impose sanctions if those authorities make unlawful or illegitimate decisions.

Now, please rate how the following items are consistent with this definition. In other words, an individual supporting institutional accountability would score higher than an individual not supporting institutional accountability. Remember that reverse items are marked by * and for those items we expect people agreeing with institutional accountability to score *lower* than individuals not agreeing with institutional accountability. Use a scale from 1 (*not consistent at all with institutional accountability*) to 7 (*highly consistent with institutional accountability*).

1. * The President should be able to implement any law, without discussing them in the Congress.
2. The Congress should discuss the laws proposed by the President.
3. The Congress or the President should not interfere with the Judicial system in the US.
4. The US should have a Congress with the ability to sanction the President when making unlawful decisions.

**Respect for liberties and freedom:** This dimension refers to the existence of liberties and freedom that are fundamental for democracy because they allow people to make informed and free decisions when deliberating or participating in politics.

Now, please rate how the following items are consistent with this definition. In other words, an individual supporting respect for liberties and freedom would score higher than an individual not supporting respect for liberties and freedom. Remember that reverse items are marked by * and for those items we expect people agreeing with respect for liberties and freedom to score *lower* than individuals not agreeing with respect for liberties and freedom. Use a scale from 1 (*not consistent at all with respect for liberties and freedom*) to 7 (*highly consistent with respect for liberties and freedom*).

1. * People should not be free to practice the religion they want.
2. The media should report the news without censorship.
3. People should be able to say what they want without censorship.
4. People should be able to access and use internet without censorship.
5. * Human rights organizations should not be allowed to operate without interference.

**Participation in politics:** This dimension refers to the right and freedom to participate in different forms of institutional and noninstitutional politics.

Now, please rate how the following items are consistent with this definition. In other words, an individual supporting participation in politics would score higher than an individual not supporting participation in politics. Remember that reverse items are marked by * and for those items we expect people agreeing with respect for liberties and freedom to score *lower* than individuals not agreeing with respect for liberties and freedom. Use a scale from 1 (*not consistent at all with respect for liberties and freedom*) to 7 (*highly consistent with respect for liberties and freedom*).

1. * Elections should not be held regularly to elect members of the Congress and the President.
2. People should be able to freely join political parties if they want.
3. * People should not be able to protest if they do not agree with the authorities.
4. People should be able to freely join opposition parties if they want.
5. People should be able to run for public office if they want.

## Table S20. Results from Pilot Study

|  | M | SD |
| --- | --- | --- |
| Institutional Accountability | | |
| * The President should be able to implement any law, without discussing them in the Congress. | 2.34 | 1.80 |
| The Congress should discuss the laws proposed by the President. | 5.72 | 1.34 |
| The Congress or the President should not interfere with the Judicial system in the US. | 3.89 | 1.91 |
| The US should have a Congress with the ability to sanction the President when making unlawful decisions. | 5.67 | 1.49 |
| Respect for Liberties and Freedom | | |
| * People should not be free to practice the religion they want. | 2.49 | 2.09 |
| The media should report the news without censorship. | 5.24 | 1.80 |
| People should be able to say what they want without censorship. | 5.19 | 1.65 |
| People should be able to access and use internet without censorship. | 5.46 | 1.54 |
| * Human rights organizations should not be allowed to operate without interference. | 2.92 | 1.90 |
| Participation in Politics | | |
| * Elections should not be held regularly to elect members of the Congress and the President. | 2.70 | 1.99 |
| People should be able to freely join political parties if they want. | 6.00 | 1.26 |
| * People should not be able to protest if they do not agree with the authorities. | 2.62 | 1.86 |
| People should be able to freely join opposition parties if they want. | 5.73 | 1.37 |
| People should be able to run for public office if they want. | 5.69 | 1.44 |
